# High- and ultrahigh-field magnetic resonance imaging of naïve, injured and scarred vocal fold mucosae in rats

**DOI:** 10.1242/dmm.026526

**Published:** 2016-11-01

**Authors:** Ayami Ohno Kishimoto, Yo Kishimoto, David L. Young, Jinjin Zhang, Ian J. Rowland, Nathan V. Welham

**Affiliations:** 1 Department of Surgery, Division of Otolaryngology, University of Wisconsin School of Medicine and Public Health, Madison, WI 53792, USA; 2 Department of Radiology andCenter for Magnetic Resonance Research, University of Minnesota–Twin Cities, Minneapolis, MN 55455, USA; 3 Department of Entomology, University of Wisconsin–Madison, Madison, WI 53706, USA

**Keywords:** Fibrosis, Hemorrhage, Larynx, MRI, Tissue repair, Voice, Wound healing

## Abstract

Subepithelial changes to the vocal fold mucosa, such as fibrosis, are difficult to identify using visual assessment of the tissue surface. Moreover, without suspicion of neoplasm, mucosal biopsy is not a viable clinical option, as it carries its own risk of iatrogenic injury and scar formation. Given these challenges, we assessed the ability of high- (4.7 T) and ultrahigh-field (9.4 T) magnetic resonance imaging to resolve key vocal fold subepithelial tissue structures in the rat, an important and widely used preclinical model in vocal fold biology. We conducted serial *in vivo* and *ex vivo* imaging, evaluated an array of acquisition sequences and contrast agents, and successfully resolved key anatomic features of naïve, acutely injured, and chronically scarred vocal fold mucosae on the *ex vivo* scans. Naïve lamina propria was hyperintense on T1-weighted imaging with gadobenate dimeglumine contrast enhancement, whereas chronic scar was characterized by reduced lamina propria T1 signal intensity and mucosal volume. Acutely injured mucosa was hypointense on T2-weighted imaging; lesion volume steadily increased, peaked at 5 days post-injury, and then decreased – consistent with the physiology of acute, followed by subacute, hemorrhage and associated changes in the magnetic state of hemoglobin and its degradation products. Intravenous administration of superparamagnetic iron oxide conferred no T2 contrast enhancement during the acute injury period. These findings confirm that magnetic resonance imaging can resolve anatomic substructures within naïve vocal fold mucosa, qualitative and quantitative features of acute injury, and the presence of chronic scar.

## Abstract (precis)

**Summary:** Magnetic resonance imaging is shown to allow the nondestructive assessment of acute injury and scar formation in vocal fold mucosa, as demonstrated *ex vivo* using a preclinical rat model.

## INTRODUCTION

The vocal fold mucosae are a pair of biomechanically exquisite, voice-generating tissues housed in the larynx. Clinically, vocal fold mucosal integrity is evaluated using direct or indirect laryngoscopy (Rosen and Murry, 2000; Sulica, 2013). Epithelial lesions can be identified visually; however, subepithelial lesions can be difficult to differentiate based on external appearance alone and so are typically inferred from their impact on vocal fold oscillation during voicing (Rosen et al., 2012). This is particularly true in the case of vocal fold scar, which does not alter the mucosal edge contour to the extent of other benign subepithelial lesions (Dailey and Ford, 2006). Pathological diagnosis using mucosal biopsy carries a risk of iatrogenic injury, scar formation and chronic dysphonia, and so is generally reserved for cases involving clinical suspicion of a malignant neoplasm. Consequently, most subepithelial lesions are not definitively diagnosed until the time of surgical resection and pathology readout. There is therefore a need for improved nondestructive assessment of the vocal fold mucosae, to assist with provisional diagnosis, treatment planning and disease monitoring.

A number of nondestructive imaging modalities have been proposed in an attempt to better evaluate the vocal fold mucosa *in situ*. Optical coherence tomography (OCT) and high-frequency (>30 kHz) ultrasound provide high-resolution, cross-sectional imaging of tissues and have been used to evaluate naïve, pathologic and surgically manipulated vocal fold mucosae in preclinical models and human patients (Burns et al., 2011, 2009; Coughlan et al., 2016; Huang et al., 2007; Walsh et al., 2008; Wong et al., 2005). Imaging data are available in real time; however, with the exception of long-range OCT (Coughlan et al., 2016; Vokes et al., 2008), these techniques require endolaryngeal placement of an imaging probe used in contact or near-contact mode, have limited depth penetration and do not provide full anatomic context for the region of interest. Magnetic resonance imaging (MRI) is an alternative technology that allows high-resolution, high-contrast imaging of whole tissues. Unlike other whole-specimen imaging techniques such as X-ray and computed tomography, MRI does not deliver ionizing radiation. It does not require placement of an imaging probe, is not limited to cross-sectional imaging and can be used to acquire three-dimensional data. Clinical MRI is generally performed using a field strength of 1.5-3.0 T; however, preclinical MR instruments are commercially available with field strengths as high as 21.1 T (Schepkin et al., 2010; Sharma, 2009; Sharma and Sharma, 2011), providing spatial resolution comparable with the ∼10-50 µm reported for OCT and high-frequency ultrasound. A previous report of ultrahigh-field (11.7 T) imaging of *ex vivo* ferret and canine larynges showed clear identification of basic vocal fold sub-structures, experimentally induced scar, and injected biomaterials at 39 µm^2^/pixel resolution (Herrera et al., 2009). This proof-of-concept study demonstrated the potential of MRI for the nondestructive characterization of vocal fold subepithelial tissue changes.

Here, to expand on this previous work, we assessed the ability of high- and ultrahigh-field MRI to resolve key vocal fold tissue structures in the rat; an important and widely used preclinical model in vocal fold biology (Riede et al., 2011; Tateya et al., 2006; Welham et al., 2015). We conducted serial *in vivo* and *ex vivo* imaging, evaluated an array of acquisition sequences and contrast agents, and successfully characterized features of both acute vocal fold injury and chronic vocal fold scar.

## RESULTS

### MRI of the naïve rat larynx

**Fig. 1.**
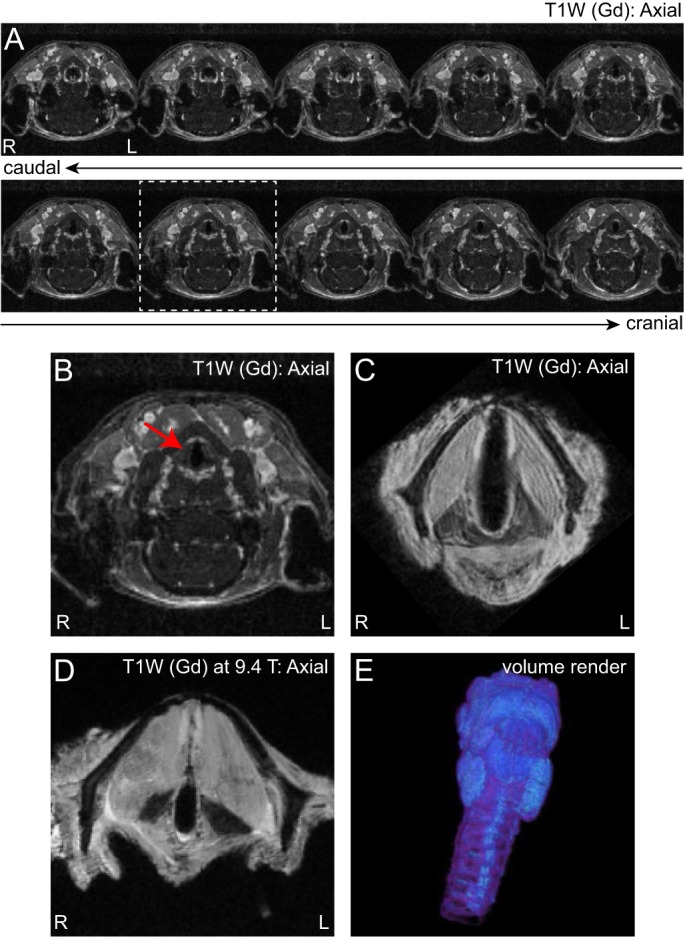
**MRI of the naïve rat larynx, *in vivo* and *ex vivo*.** (A) T1-weighted (T1W) serial axial images of the rat neck, acquired *in vivo* at 4.7 T using intravenous contrast enhancement. (B) Enlarged image of the region indicated by the dashed square in A. The red arrow indicates the larynx. (C) T1W axial image of the rat larynx, acquired *ex vivo* at 4.7 T using immersion contrast enhancement. (D) T1W axial image of the naïve rat larynx, acquired *ex vivo* at 9.4 T using immersion contrast enhancement. (E) Pseudocolored volume render of the rat larynx, generated with data from an *ex vivo* scan at 4.7 T using immersion contrast enhancement. Data represent *n*=5 animals per *in vivo*/*ex vivo* condition at 4.7 T (A-C,E) and *n*=2 animals at 9.4 T (D). Gd, gadobenate dimeglumine contrast agent; R, right; L, left.

To our knowledge, despite the availability of human and large animal data (Chen et al., 2012; Herrera et al., 2009), there are no previous reports of MRI of the rat larynx. We therefore began by imaging naïve rats *in vivo* and naïve rat larynges *ex vivo* to evaluate the ability of MRI to resolve key anatomic structures at 4.7 and 9.4 T. T1-weighted (T1W) *in vivo* imaging of the rat neck with intravenous gadobenate dimeglumine (Gd) contrast enhancement provided clear identification of the glottis and some cartilaginous structures at 273 µm^3^/voxel resolution, but did not resolve individual cartilages, muscles or sub-structures within the vocal fold mucosae (Fig. 1A,B). Overnight (∼6 h) T1W imaging of *ex vivo* naïve larynges following 10 days of Gd immersion contrast enhancement allowed identification of hyperintense vocal fold mucosae, individual intrinsic laryngeal muscles and hypointense laryngeal cartilages (Fig. 1C). These structures were identified at 41 µm^3^/voxel resolution; we obtained comparable resolution of key laryngeal sub-structures with 10 min T1W scans at 9.4 T (Fig. 1D). The acquisition of three-dimensional data allowed precise volume rendering of all laryngeal structures (Fig. 1E).

### Evaluation of acute vocal fold injury with intravenous SPIO

Vocal fold mucosal injury in the rat model results in peak cellular infiltration at 5 days post-injury (Ling et al., 2010a). This infiltrating population includes monocyte lineage cells, such as fibrocytes and macrophages (Ling et al., 2010b). As proinflammatory macrophages are known to engage in iron uptake and sequestration (Cairo et al., 2011), and because paramagnetic iron causes shortening of T2 relaxation time on MRI (Chen et al., 1999), we evaluated whether the intravenous delivery of superparamagetic iron oxide (SPIO) nanoparticles could enhance MRI contrast of the acutely injured vocal fold mucosa. This approach has been successfully used to study macrophage infiltration of both central and peripheral nervous system injuries in experimental models (Bendszus and Stoll, 2003; Kleinschnitz et al., 2003; Stoll et al., 2004), as well as identification of liver and spleen lesions on clinical MRI (as most circulating SPIO is eventually phagocytized by Kupffer cells in the liver and red pulp macrophages in the spleen) (Chen et al., 1999; Schuhmann-Giampieri, 1993).

**Fig. 2.**
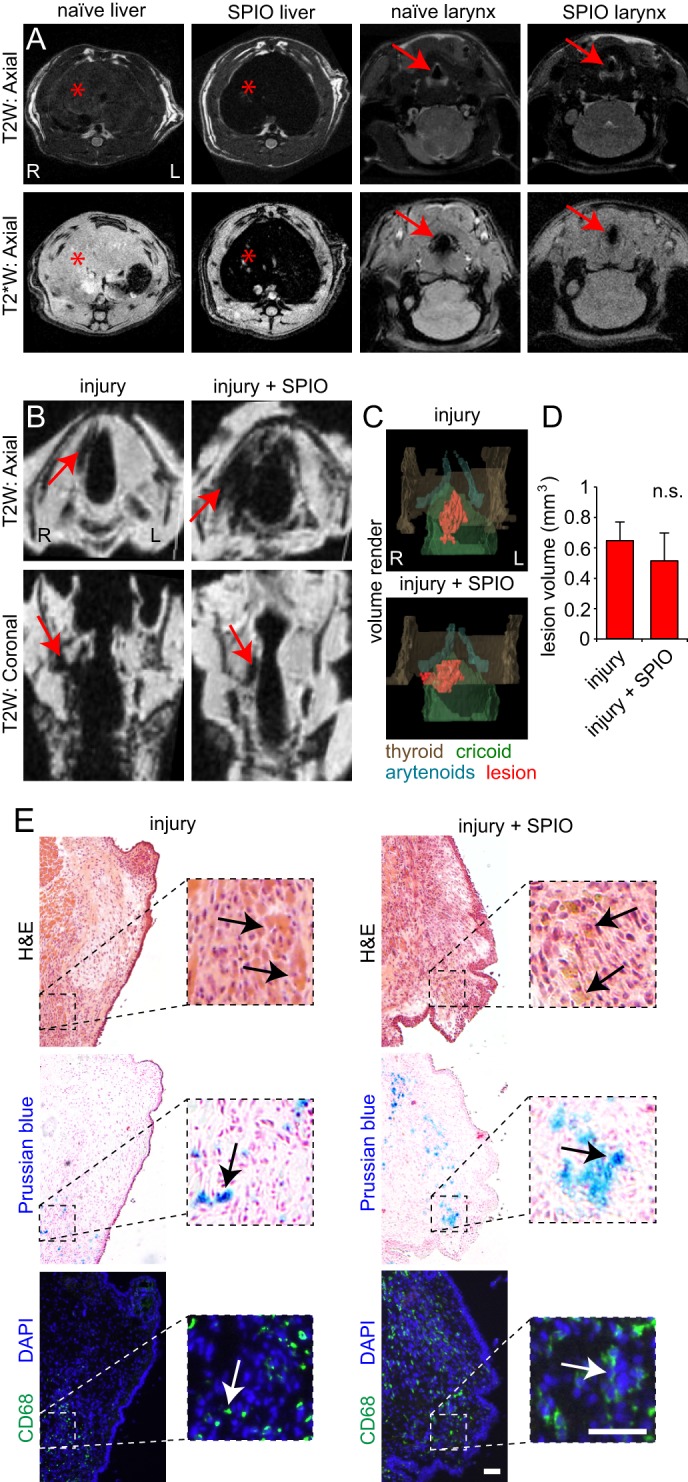
**Superparamagnetic iron oxide (SPIO) contrast enhancement of acute vocal fold injury.** (A) T2- and T2*-weighted (T2W, T2*W) coronal images of the rat abdomen and neck, acquired *in vivo* at 4.7 T with and without intravenous SPIO contrast enhancement. Red asterisks indicate livers, red arrows indicate larynges. (B) T2W axial and coronal images of the rat larynx, 5 days following right-sided vocal fold mucosal injury. Images were acquired *ex vivo* at 4.7 T, with and without (pre-explant) intravenous SPIO contrast enhancement. Red arrows indicate hypointense mucosal lesions. (C) Pseudocolored volume renders of the vocal fold mucosal lesions shown in B. Lesions are red; thyroid (brown), cricoid (green) and arytenoid (cyan) cartilages are shown for anatomic orientation. (D) Effect of contrast enhancement on vocal fold mucosal lesion volume (mean±s.e.m.); n.s., no significant difference (*P*>0.01), calculated using a Student's *t*-test. (E) H&E-, Prussian Blue- and CD68-stained vocal fold coronal sections, 5 days following mucosal injury. Black arrows indicate blood (red) and hemosiderin (brown) in the H&E-stained sections and ferric iron (blue) in the Prussian Blue-stained sections; white arrows indicate CD68^+^ cells (green) in the immunosections (nuclei are counterstained blue). Scale bars: 100 µm. Data represent *n*=5 animals per experimental condition in A-E, with the exception of the injury+SPIO images and render in panels B and C; these data represent *n*=2/5 animals in which contrast enhancement was associated with larger lesion volumes. R, right; L, left.

We created unilateral vocal fold mucosal injuries, injected intravenous SPIO 4 days post-injury and performed *in vivo* followed by *ex vivo* imaging 5 days post-injury. Non-SPIO-treated rats served as controls. Abdominal scans showed liver hypointensity on T2W and T2*W images following SPIO administration, confirming successful nanoparticle migration and uptake by Kupffer cells *in vivo* (Fig. 2A). Despite this evidence of cell-mediated modulation of liver signal intensity, we were unable to resolve the vocal fold lesions *in vivo*, owing to insufficient imaging resolution (Fig. 2A). Follow-up T2W imaging of the explanted larynges *ex vivo* resulted in clear identification of the unilateral lesions as hypointense tissue regions, irrespective of the presence or absence of SPIO (Fig. 2B). SPIO contrast enhancement was associated with larger lesion volumes in certain cases (Fig. 2B,C); however, quantitative analysis of lesion volumes showed no overall advantage with SPIO (*P*>0.01; Fig. 2D). We identified residual hemorrhage and hemosiderin on hematoxylin and eosin (H&E) staining, ferric iron on Prussian Blue staining, and CD68^+^ macrophages on immunostaining (Fig. 2E). These features were present in both the presence and absence of SPIO.

### Characterization of the acute vocal fold injury time course

**Fig. 3.**
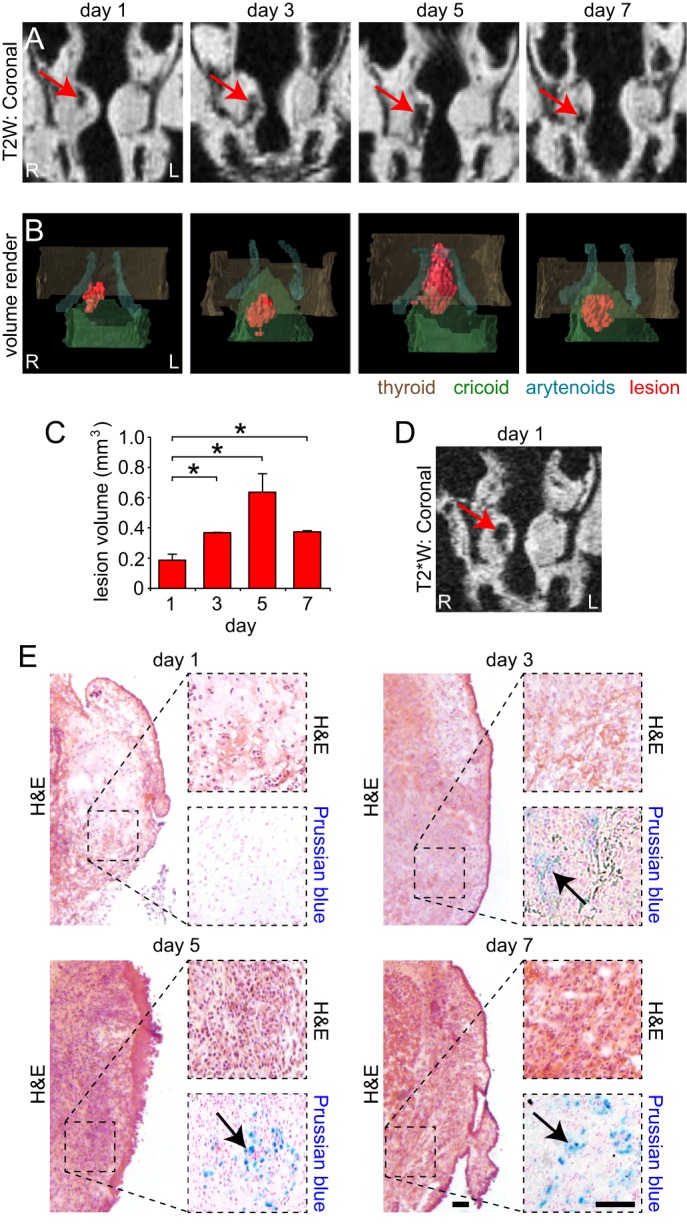
**Characterization of the acute vocal fold injury time course.** (A) T2-weighted (T2W) coronal images of the rat larynx, 1-7 days following right-sided vocal fold mucosal injury. Images were acquired *ex vivo* at 4.7 T. Red arrows indicate hypointense mucosal lesions. (B) Pseudocolored volume renders of the vocal fold mucosal lesions shown in A. Lesions are red; thyroid (brown), cricoid (green) and arytenoid (cyan) cartilages are shown for anatomic orientation. (C) Change in vocal fold mucosal lesion volume, 1-7 days post-injury (mean±s.e.m.); *P<0.01 compared with day 1, calculated using one-way ANOVA. (D) T2*W coronal image of the rat larynx, 1 day following right-sided vocal fold mucosal injury. The image was acquired *ex vivo* at 4.7 T and is from the same 1 day post-injury larynx shown in A. The red arrow indicates a hypointense mucosal lesion. (E) H&E- and Prussian Blue-stained vocal fold coronal sections, 1-7 days following mucosal injury. Black arrows indicate ferric iron (blue). Scale bars: 100 µm. Data represent *n*=5 animals per experimental time point. R, right; L, left.

Given that intravenous SPIO conferred no benefit during *ex vivo* T2W imaging of acute vocal fold injury at 5 days post-injury, we proceeded to characterize the acute injury time course without SPIO. We created unilateral injuries as described above and performed *ex vivo* imaging at 1, 3, 5 and 7 days post-injury. The hypointense vocal fold lesions were clearly identified with T2W imaging at each time point (Fig. 3A). Lesion volume steadily increased over the first 5 days, peaked at day 5, and decreased on day 7 post-injury (*P*<0.01; Fig. 3B,C). We acquired additional T2*W images 1 day post-injury that also showed tissue hypointensity at the lesion site (Fig. 3D), consistent with acute hemorrhage (Bradley, 1993). Using histology, we confirmed the presence of acute, and then resolving, hemorrhage over the experimental time course (Fig. 3E). Ferric iron was first detected at 3 days post-injury, and showed increased abundance at 5 and 7 days post-injury (Fig. 3E).

### Characterization of vocal fold scar

**Fig. 4.**
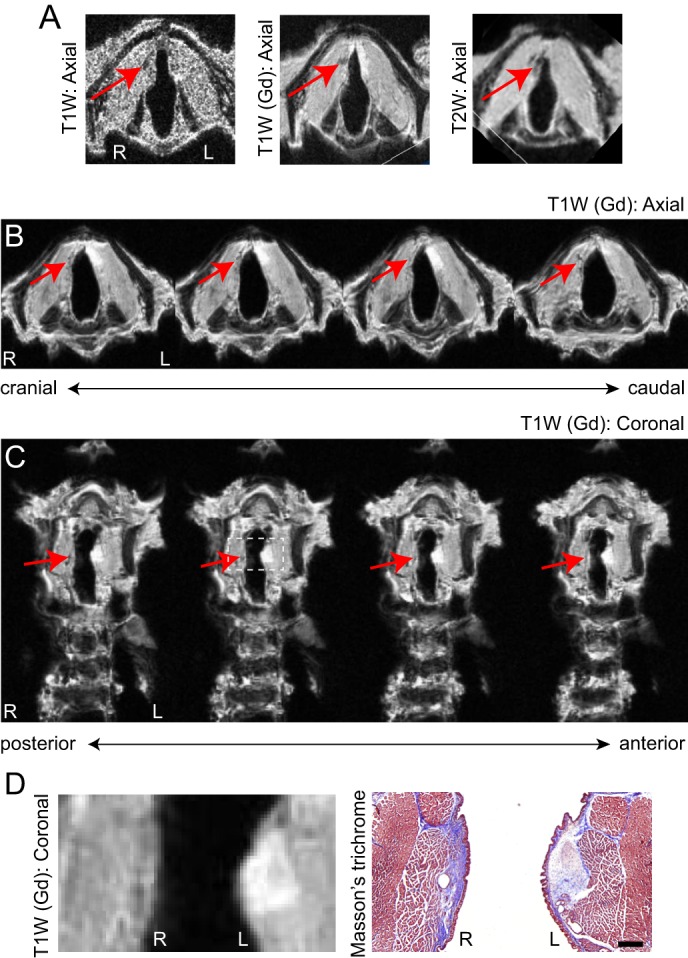
**Characterization of vocal fold scar.** (A) T1- and T2-weighted (T1W, T2W) axial images of the rat larynx, 2 months following right-sided vocal fold mucosal injury. Images were acquired *ex vivo* at 4.7 T; T1W images were acquired with (center) and without (left) immersion contrast enhancement. (B) T1W serial axial images of the rat larynx, 2 months following right-sided vocal fold mucosal injury. Images were acquired *ex vivo* at 4.7 T using immersion contrast enhancement. (C) Resliced serial coronal images of the larynx shown in B. (D) Enlarged image of the region indicated by the dashed square in C (left); Masson's Trichrome-stained section of the same larynx (right). Scale bar: 300 µm. Red arrows indicate hypointense scar tissue. Data represent *n*=5 animals. Gd, gadobenate dimeglumine contrast agent; R, right; L, left.

Mature vocal fold scar appears ∼2 months following mucosal injury in the rat (Tateya et al., 2005; Welham et al., 2015). To evaluate our ability to resolve vocal fold scar tissue with MRI, we created unilateral vocal fold mucosal injuries and performed *ex vivo* imaging following this 2-month scar maturation period. The scarred mucosa appeared as a hypointense and volumetrically deficient region on T1W and T2W images (Fig. 4A); the greatest contrast with the hyperintense naïve mucosa was obtained with a T1W imaging sequence following Gd immersion (Fig. 4A-C). Post-scan validation of scar localization using histology confirmed the hallmark features of dense collagen deposition and overall tissue contraction (Fig. 4D).

## DISCUSSION

Improved nondestructive and noninvasive imaging of the vocal fold mucosa would transform both preclinical research and clinical practice. MRI technology continues to advance at a rapid pace and is increasingly capable of meeting this imaging need. High- and ultrahigh-field instruments are commercially available; proof-of-concept and experimental animal studies conducted with such instruments can help guide the development of future clinical protocols. To this end, our goal was to further develop the applicability of MRI-based imaging of the vocal fold mucosa, using a preclinical rat model. We focused on naïve, acutely injured and chronically scarred vocal fold mucosa, as wound healing is relatively well conserved in humans and rats (Hirano et al., 2009; Tateya et al., 2005), scarring is primarily a subepithelial pathology (Gurtner et al., 2008; Martin, 1997), and because vocal fold scar can be challenging to assess using traditional imaging modalities (Dailey and Ford, 2006). Our data show that high- and ultrahigh-field MRI can resolve key anatomic features of the naïve rat larynx and its vocal fold mucosae, qualitative and quantitative elements of the acute injury phase, and the presence of chronic scar. This imaging was most successful with *ex vivo* specimens.

We resolved key anatomic features of the naïve and scarred vocal fold mucosae *ex vivo* using T1W imaging and Gd contrast enhancement. This paramagnetic, extracellular contrast agent distributes within the intravascular and interstitial spaces and differentially alters tissue contrast by shortening T1 relaxation time (Weinmann et al., 1984). At both 4.7 and 9.4 T, the naïve vocal fold mucosa – specifically its lamina propria – was hyperintense on T1W imaging compared with the adjacent intrinsic laryngeal muscles and cartilages. The vocal fold lamina propria is viscoelastic (Chan and Titze, 1999), anisotropic (with primary alignment of fibrous matrix proteins along its anterior–posterior plane) (Julias et al., 2013; Kelleher et al., 2014), and consists of an extracellular matrix rich in hydrophilic glycans such as hyaluronic acid (Gray et al., 1999). The tendency of these glycans to bind water makes the lamina propria ideal for MR signal enhancement (Sadeghi et al., 2003). Compared with the naïve condition, vocal fold scar was characterized by a sharp decrease in lamina propria T1 signal intensity and overall mucosal volume, consistent with previous *ex vivo* ferret data collected at 11.7 T (Herrera et al., 2009). These MRI features corresponded to increased collagen abundance and tissue contraction; previous work with the rat model has also shown loss of hyaluronic acid (Tateya et al., 2005).

We evaluated the acute injury response using T2W imaging and observed peak hypointense lesion volume at day 5. T2W sequences are highly sensitive to the magnetic state of hemoglobin and its degradation products within an acute or subacute hemorrhage; in certain clinical scenarios such as brain hemorrhage, MRI is used to help stage the injury (Bradley, 1993). Classically, early acute hemorrhage is populated by a mixture of oxy- and deoxyhemoglobin-containing red blood cells. Both forms contain heme iron in its ferrous state; however, oxyhemoglobin is diamagnetic whereas deoxyhemoglobin – where the iron atoms contain four unpaired electrons – is paramagnetic. As the hemorrhage begins to mature, oxyhemoglobin is converted to deoxyhemoglobin, which in turn is oxidized to form methemoglobin (containing paramagnetic ferric heme iron atoms possessing five unpaired electrons). Finally, red blood cell lysis occurs, spilling methemoglobin into the extracellular space where it is further denatured into a range of iron-containing hemichromes and targeted for phagocytosis by macrophages. Macrophages accumulate iron and deposit it within the iron storage protein ferritin, which itself might degrade into hemosiderin. These physiologic changes typically yield maximum shortening of T2 relaxation time at the late-acute and subacute phases, when paramagnetic deoxyhemoglobin and methemoglobin predominate at the injury site (Bradley, 1993). Moreover, iron cores within ferritin and hemosiderin are superparamagnetic and possess T2 and T2* shortening properties similar to those of SPIO.

Our findings in rat vocal fold mucosa are consistent with these principles. Deoxyhemoglobin seemed to be present by day 1 post-injury, indicated by the initial hypointense lesion on T2W imaging, the acute hemorrhage on H&E staining, and the absence of ferric iron on Prussian Blue staining. Ferric iron was first detected at day 3, suggesting early oxidation of heme iron and, therefore, the presence of methemoglobin. Peak lesion volume at day 5 corresponded to a marked increase in ferric iron signal intensity, as well as the identification of CD68^+^ macrophages and intracellular hemosiderin – consistent with further oxidative denaturation and methemoglobin accumulation, in addition to hemichrome deposition and the beginning of phagocytosis. The decrease in lesion volume at day 7, combined with the persistence of ferric iron, suggests an ongoing progression from paramagnetic methemoglobin to hemichrome formation.

We were unable to clearly identify macrophage infiltration of the acute injury site using SPIO contrast enhancement at 5 days post-injury. Whereas certain SPIO-treated animals – such as the case shown in Fig. 2 – exhibited increased lesion volumes, greater ferric iron abundance and greater CD68^+^ macrophage infiltration, there was no significant group difference in lesion volume between animals with and without SPIO. This finding might have resulted from the accumulation of multiple paramagnetic substances (endogenous deoxyhemoglobin and methemoglobin, as well as exogenous SPIO) at the lesion site at day 5, causing a saturation of T2 signal loss. It is well known that acute and subacute hemorrhage exhibit strong T2 hypointensity; hemorrhage can also contribute to regional magnetic non-uniformity and blooming artifact (Bradley, 1993). Therefore, despite peak cellular infiltration at day 5 (Ling et al., 2010a,b), the presence of SPIO might have had minimal impact on overall T2 hypointensity, compared with that of endogenous heme iron.

The rat vocal fold mucosa has dimensions of ∼1.0-1.4 mm along its anterior–posterior plane, ∼1.0 mm along its superior–inferior plane, and ∼0.2-0.5 mm along its medial–lateral plane, yielding a volume of less than 0.2 mm^3^ (Kurita et al., 1983; Ling et al., 2010a). Despite this relatively small volume, we were able to optimize MRI protocols for naïve, acutely injured and chronically scarred mucosae at 4.7 T by using an *ex vivo* tissue setup, immersion contrast enhancement, appropriate image weighting and extended acquisition times. These protocols yielded useful data at 41 µm^3^/voxel resolution, comparable with the 39 µm^2^/pixel resolution reported by Herrera et al. (2009) in (substantially larger) *ex vivo* ferret and dog larynges. Despite this progress *ex vivo*, practical implementation of MRI for the assessment of vocal fold sub-structures in experimental animals and human patients requires high-resolution *in vivo* scanning, which in turn requires higher field strengths, improved signal-to-noise ratios, and shorter acquisition times. Towards this end, we obtained equivalent spatial resolution and image quality with substantially less acquisition time when using a 9.4 T magnet. Even higher-resolution MRI is feasible in rodents *in vivo* at 16.4 and 21.1 T (Schepkin et al., 2010; Shajan et al., 2012; Ullmann et al., 2013, 2014); *in vivo* human MRI has been reported with field strengths as high as 9.4 T (Pohmann et al., 2016). The use of a purpose-designed surface coil could provide further improvements in signal-to-noise ratio (McArdle et al., 1986). Additional considerations when translating this approach include the use of respiratory gating and (in the case of humans) behavioral strategies to reduce motion artifacts associated with breathing, swallowing and coughing (Ehman et al., 1984; Nygren et al., 2016).

In summary, our data demonstrate the potential of current preclinical MRI technology for the assessment of vocal fold subepithelial tissue changes in the rat model. Further progress, technology development and regulatory approvals might reduce the number of animals needed for preclinical studies as the vocal fold injury response and disease progression could be monitored using *in vivo* serial scans. Moreover, next generation high- and ultrahigh-field MR instruments might one day assist clinicians and surgeons as they evaluate subepithelial changes to the vocal fold mucosa, consider differential diagnoses and engage in treatment decision making.

## MATERIALS AND METHODS

### Animals

Four-month-old Fisher 344 male rats (total *n*=47; Charles River, Wilmington, MA) were used for all experiments. All *in vivo* work was conducted in accordance with the Public Health Service Policy on Humane Care and Use of Laboratory Animals and the Animal Welfare Act (7 U.S.C. et seq.). All protocols were approved by the Institutional Animal Care and Use Committee of the University of Wisconsin–Madison.

### Vocal fold mucosal injury

Unilateral vocal fold mucosal injuries were created as previously reported (Ling et al., 2010a; Tateya et al., 2005). Briefly, rats underwent anesthesia induction using isoflurane (2-3%, mixed with 100% oxygen and delivered via induction chamber at 0.8-1.5 l/min) followed by maintenance using an intraperitoneal injection of ketamine hydrochloride (90 mg/kg) and xylazine hydrochloride (9 mg/kg). Atropine sulfate (0.05 mg/kg) was also injected intraperitoneally to reduce the secretion of saliva and sputum in the laryngeal lumen. The anesthetized rats were placed on an operating platform and a 1 mm diameter steel wire laryngoscope was inserted to facilitate vocal fold visualization. A 1.9 mm diameter, 25° rigid endoscope (Richard Wolf, Vernon Hills, IL) connected to an external light source and video monitor was used for surgical monitoring. The right vocal fold mucosa was stripped using a 25 gauge needle.

Animals in the acute injury experiment (*n*=5 per time point and SPIO condition) underwent *in vivo* MRI, followed by euthanasia and tissue harvest for *ex vivo* MRI, at 1, 3, 5 and 7 days post-injury; animals in the chronic scar experiment (*n*=5) underwent euthanasia and tissue harvest for *ex vivo* MRI at 2 months post-injury.

### Magnetic resonance imaging

MRI was primarily performed in a 4.7 T instrument (Agilent Technologies, Santa Clara, CA) using a 210 mm bore magnet and standard volume coil. An additional high-field scan of the naïve larynx was performed in a 9.4 T instrument (Magnex Scientific, Yarnton, UK) using a 310 mm bore magnet and standard volume coil. VnmrJ software (Agilent Technologies) was used for instrument control and data acquisition.

*In vivo* scans were performed under isoflurane maintenance anesthesia (1%, mixed with 100% oxygen and delivered via nose cone at 1.0 l/min). Respiratory rate was monitored using a SAII model 1030 system (SA Instruments, Stony Brook, NY). For experimentally naïve rats, we began by acquiring T1W neck images without contrast enhancement. Next, to reduce longitudinal relaxation time and evaluate its effect on tissue contrast, we injected intravenous Gd contrast agent (0.5 mmol/kg MultiHance, Bracco Imaging, Princeton, NJ) and repeated the T1W acquisition sequence. For rats in the acute injury experiment, we acquired T2W and T2*W abdominal and neck images at each post-injury time point. To reduce T2 relaxation time and evaluate its effect on tissue contrast, we injected a subset of rats in the 5 day post-injury group with intravenous SPIO contrast agent [200 µmol Fe/kg Ferex (∼5 nm iron core size, ∼50-150 nm colloidal matrix size), BioPal, Worcester, MA], 24 h before image acquisition.

*Ex vivo* scans were performed as follows. For experimentally naïve rats and those in the chronic scar experiment, larynges were explanted and stored in 4% paraformaldehyde (PFA) prior to image acquisition. Most T1W images were acquired following immersion in 5 mM Gd contrast agent (MultiHance) in 4% PFA for 10 days. For comparison, a small number of non-contrast enhanced images were acquired prior to immersion in Gd. For rats in the acute injury experiment, T2W and T2*W images were acquired immediately following the *in vivo* scans and laryngeal explant. All *ex vivo* samples were blotted to remove surface fluid and then suspended in liquid perfluorocarbon prior to scanning.

We used the following acquisition protocols at 4.7 T: (1) T1W gradient echo, *in vivo* [15/5 ms repetition/echo times, 65° flip angle, 256×128×128 matrix, 70×35×35 mm field-of-view (FOV)]; (2) T1W gradient echo, *ex vivo* (50/6.5 ms repetition/echo times, 65° flip angle, 512×256×256 matrix, 30×15×15 mm FOV); (3) T2W gradient echo (93/12 ms repetition/echo times, 45° flip angle, 128×128×128 matrix, 18×12×12 mm FOV); (4) T2*W gradient echo (70/20 ms repetition/echo times, 20° flip angle, 128×128×128 matrix, 18×12×12 mm FOV). We used the following acquisition protocol at 9.4 T: T1W gradient echo, *ex vivo* (8.5/4 ms repetition/echo times, 8° flip angle, 256×256×256 matrix, 12×20×12 mm FOV).

Scan data were analyzed using ImageJ (Schneider et al., 2012). Volume rendering and volume measurements were performed using OsiriX 6.0 (Pixmeo, Bernex, Switzerland) and Amira 5.2 (Visage Imaging, Berlin, Germany).

### Histology and immunohistochemistry

All scanned larynges were processed for histology and/or immunohistochemistry (IHC). Using whole laryngeal blocks, 6 µm frozen serial sections were prepared in the coronal plane. Sections that included the midmembranous vocal folds were retained for staining. Routine H&E staining was used to evaluate cell and tissue morphology; routine Masson's Trichrome staining was used to evaluate collagen deposition. Prussian Blue staining was used to detect ferric iron, as follows: sections were immersed in a 1:1 cocktail of 20% hydrochloric acid and 10% potassium ferrocyanide for 20 min, rinsed in deionized water, counterstained with Nuclear Fast Red solution (Newcomer Supply, Middleton, WI) for 5 min, dehydrated, and coverslipped.

Sections intended for IHC were fixed using acetone for 10 min, washed with phosphate-buffered saline (PBS), and blocked using 5% BSA (Sigma, St Louis, MO) for 60 min. Next, sections were sequentially incubated with mouse anti-rat CD68, clone ED1 (1:750; MCA341, AbD Serotec, Raleigh, NC) for 90 min, followed by Alexa Fluor 488 goat anti-mouse IgG (1:800; A11001, Life Technologies, Grand Island, NY) for 60 min, counterstained with DAPI (1:5000; Life Technologies) for 5 min, and coverslipped. Rat spleen was used as a positive control. Negative control sections, stained without the primary antibody, ensured each immunosignal was specific to the intended antigen.

Images were captured using a microscope with both bright field and fluorescent capabilities (E-600; Nikon, Melville, NY), equipped with a digital microscopy camera (DP-71; Olympus, Center Valley, PA).

### Statistics

Given the absence of existing MRI data characterizing lesion volumes following acute vocal fold injury, we powered this experiment using histologic measures of rat vocal fold mucosal cross-sectional area at our post-injury time points of interest (Ling et al., 2010a). Based on these data, we estimated that *n*=5 animals per time point would allow detection of a >1 s.d. shift in mean lesion volume with 80% power. Animals were not randomized. All image analysis procedures were performed on blinded samples.

No data points were removed prior to statistical analysis. Data were evaluated for normality and equality of variance using visual inspection of raw data plots and Levene's test. The data did not meet the normality assumption and were therefore rank-transformed prior to additional testing. Lesion volume data were analyzed using a Student's *t-*test for the comparison of injury and injury+SPIO conditions at 5 days post-injury (Fig. 2D), and one-way analysis of variance (ANOVA) for assessment of the acute post-injury time course (Fig. 3C). In the ANOVA model, as the *F* test showed a significant difference across time points, Fisher's protected least significant difference method was used for planned pairwise comparisons. A type I error rate of 0.01 was used for all statistical testing; all *P*-values were two-sided.
